# Molecular Hydrogen Mitigates Performance Decrement during Repeated Sprints in Professional Soccer Players

**DOI:** 10.3390/nu14030508

**Published:** 2022-01-25

**Authors:** Michal Botek, Deepesh Khanna, Jakub Krejčí, Michal Valenta, Andrew McKune, Barbora Sládečková, Iva Klimešová

**Affiliations:** 1 Faculty of Physical Culture, Palacký University Olomouc, 77111 Olomouc, Czech Republic; michal.botek@upol.cz (M.B.); jakub.krejci@upol.cz (J.K.); michal.valenta@upol.cz (M.V.); barbora.sladeckova@upol.cz (B.S.); iva.klimesova@upol.cz (I.K.); 2 Department of Foundational Sciences, Dr. Kiran C. Patel College of Osteopathic Medicine, Nova Southeastern University, Clearwater, FL 33759, USA; 3 Research Institute for Sport and Exercise (UCRISE), University of Canberra, Bruce, ACT 2617, Australia; andrew.mckune@canberra.edu.au; 4 Discipline of Biokinetics, Exercise and Leisure Sciences, School of Health Sciences, University of KwaZulu-Natal, Durban 4041, South Africa

**Keywords:** hydrogen-rich water, exercise, fatigue resistance, OXOPHOS, field testing

## Abstract

Hydrogen-rich water (HRW) supplementation has been shown to have an antifatigue effect across different modes of exercise. However, its effect on repeated sprint performance is unknown. The aim of this study was to assess the effect of pre-exercise HRW consumption on repeated sprint performance, lactate, and perceptual responses using a repeated sprint protocol. This randomized, double blinded, placebo controlled, crossover study included 16 professional, male soccer players aged 18.8 ± 1.2 years. Athletes performed two indoor tests, particularly 15 × 30 m track sprints interspersed by 20 s of recovery, separated by a 1-week washout period. Sprint time was measured at 15 m and 30 m. Ratings of perceived exertion were assessed immediately after each sprint, and post-exercise blood lactate concentration was measured after the last sprint. There were significantly faster sprint times after HRW consumption compared with placebo at 15 m for the 14th and 15th sprints, representing improvements in time of 3.4% and 2.7%, respectively. Sprint time at 30 m also significantly improved by 1.9% in the HRW group in the last sprint. However, neither lactate concentrations nor ratings of perceived exertion were significantly different between HRW and placebo. Pre-exercise HRW supplementation is associated with an increased ability to reduce fatigue, especially during the later stages of repeated sprint exercise.

## 1. Introduction

Although success in the majority of racquet and team sports is dominated by technical and tactical skills [1], the importance of repeated sprint ability (RSA) seems to be a crucial fitness component of soccer performance [2]. RSA involves the performance of short duration sprints (≤10 s), interspersed by short recovery periods (≤60 s) [3,4]. The inherent demands of soccer require players to have enhanced RSA. Despite this, there is limited research examining the effects of novel supplementation beverages, such as hydrogen-rich water (HRW), on RSA.

Research has shown that the average sprint distance in soccer is 10–20 m [4] and the maximum sprint distance is 40 m [5] with a typical duration of 2–3 s [6,7]. Primary factors responsible for the ability to generate and maintain a stable, high muscle power output over successive sprints include adenosine triphosphate (ATP) depletion and resynthesis rate, and neuromuscular excitability associated with disturbances in intra- and extracellular ion homeostasis [3,4,8]. However, in relation to the potential limitations of energy metabolism, ATP depletion during a single 6 s cycling sprint was reported to be negligible [9,10], whereas muscle creatine phosphate (CrP) concentration was reduced to almost 50% of its resting values and only recovered up to 69% of pre-exercise level after a 30 s recovery [10]. It is widely accepted that CrP stores represent the most immediate and powerful “energy buffer” for ATP resynthesis, especially during a single 3 s sprint, where its contribution to ATP resynthesis was estimated to be 55%, compared with anaerobic glycolysis and the aerobic metabolic pathway, which covered approximately 33% and 3% [10,11], respectively. However, with repeated sprints, there was a progressive inhibition of anaerobic glycolysis along with the reduction in the absolute contribution of CrP to ATP production [10], and aerobic mitochondrial ATP production gradually increased up to 40% [12]. Despite the aerobic energy contribution, there is no strong evidence demonstrating a positive association between maximal oxygen uptake (VO_2_max) and RSA performance [13]. Previous research reported that RSA performance was more likely affected by muscle mitochondrial respiration rate than the magnitude of VO_2_max [14].

In addition to aerobic ATP production, mitochondria also generate harmful, cytotoxic, reactive oxygen and nitrogen species (ROS/RNS) as byproducts of oxidative metabolism [15]. Overproduction of ROS/RNS was associated with attenuated mitochondrial efficiency [16], peripheral fatigue development during repeated sprints [17], post-exercise recovery delay [18], and damage to macromolecular cell structures [19].

Molecular hydrogen (H_2_) has been shown to be a healthy, safe, non-metallic gas [15] with a strong and selective antioxidative affinity to harmful ROS/RNS, specifically hydroxyl and peroxynitrite radicals [20]. However, Sim et al. [21] showed that the effect of HRW on biological antioxidant potential is age-dependent, with a significant effect of HRW only found in participants >30 years of age. Besides its antioxidative property, H_2_ has recently been suggested to have anti-inflammatory, anti-apoptotic [22], and cell signaling properties [23], as well as reduce lactate and ratings of perceived exertion (RPE) [24]. Due to its small size, H_2_ can penetrate through the cell membrane into the cellular space as well as into the mitochondria, where it helps to maintain a redox-balance state and energy production [22]. H_2_ has also been shown to stimulate mitochondrial respiration, Q-cycle [25], and oxidative ATP phosphorylation (OXOPHOS) rate [26]. Pre-exercise HRW intake or H_2_ inhalation has been shown to have an antifatigue effect across different modes of exercise, including endurance [27,28,29], strength-endurance drills [30], cycling anaerobic power output [31], maximal isokinetic muscle strength [32], and during prolonged, intermittent sprints [33]. H_2_ administration strategies varied across studies, e.g., in duration of pre-exercise administration (30 min [30], 8 h [32], 24 h [28], 7 days [29,31], 2 weeks [33], and 4 weeks [27]) and administration mode (HRW [27,28,30,31,32,33], H_2_ inhalation [29]), which complicates the comparison of the study results. However, there is a paucity of studies examining the antifatigue effect of HRW consumption on subsequent repeated sprint performance in team sport in general, and specifically in professional soccer.

Based on the increasing role of mitochondrial ATP synthesis over extended repeated sprint protocols [12] and the ability of H_2_ to increase OXOPHOS rate [25,26] we hypothesized that an acute, pre-exercise, HRW intake would have a positive effect on repeated sprint running performance, as well as lactate and RPE responses.

## 2. Materials and Methods

### 2.1. Participants

This study included 16 professional male soccer players with the following characteristics (mean ± SD): age: 18.8 ± 1.2 years (range 17 to 21 years); body mass: 74.6 ± 7.7 kg; body height: 181.9 ± 6.1 cm; body mass index: 22.5 ± 1.4 kg m^−2^; body fat = 11.0 ± 2.1%; VO_2_max = 57.2 ± 2.2 mL kg^−1^ min^−1^. All participants were healthy (self-reported), medication free, non-smokers and not taking any dietary supplements. The study was approved by the Ethics Committee of the Faculty of Physical Culture, Palacký University Olomouc (reference number 75/2017).

### 2.2. Experimental Protocol

**Figure 1:**
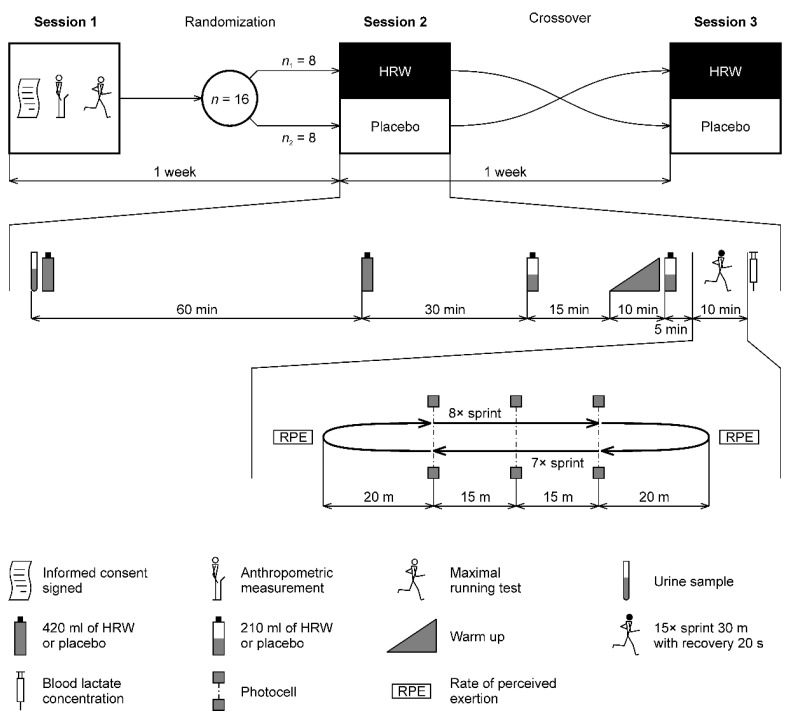
Overview of the study protocol and labeling of sessions. HRW—hydrogen-rich water.

The experimental study protocol consisted of one laboratory session and two field-testing sessions separated by a 1-week washout period (Figure 1). During the first session, participants were provided with the study information and familiarized with the testing laboratory equipment. They or their parents provided written informed consent. Anthropometric measurements were then taken, and VO_2_max determined. Participants were instructed to avoid drinking caffeine-containing beverages, such as coffee or tea, and other substances that could potentially impact the physiological, biochemical, and perceptual outcomes, 2 h before the pre-experimental testing and two experimental sessions. Participants were also instructed to avoid drinking alcohol and performing demanding physical activity for 48 h before testing. To avoid possible diurnal variation, all exercise testing was performed between 8:30 and 11:00 AM in a faculty facility as well as in the indoor athletics training center that belongs to the Athletics Club Olomouc (indoor temperature 18–20 °C). The second session proceeded 1 week after the first session. In this session, participants were randomly divided into two groups, HRW (*n*_1_ = 8) or placebo (*n*_2_ = 8), and they then followed each task scheduled in the experimental research protocol (Figure 1). Order of HRW or placebo consumption was randomized by means of lots that included an equal number of two colored strips (red and blue) to represent either HRW or placebo consumption first. Whilst blinded, participants drew one strip. There was then a 1-week washout period before the third session where the beverage consumption was reversed prior to performance of the same running protocol. The participants were instructed by the coach to consume the same diet and not make any changes in their diet over the duration of the study. The athletes ate together in the club dining room, ensuring that the type of food was the same.

### 2.3. Hydration Status

Upon arrival, participants collected a mid-stream urine sample into sterile urine containers. Hydration status was assessed based on urine specific gravity which was determined using a refractometer (SUR-NE, ATAGO, Tokyo, Japan). Between sample readings, the refractometer was recalibrated using distilled water. The urine specific gravity value is related to the density of water and is, therefore, a dimensionless number.

### 2.4. Basis Anthropometric Measurement

Participant height and body mass (nearest 0.1 kg) were determined using a digital weighing scale SOEHNLE 7307 (Leifheit, Nassau, Germany). Body fat percentage was calculated using bioelectrical impedance (Tanita BC-418 MA, Tanita, Tokyo, Japan).

### 2.5. Maximal Running Test

Each participant performed an incremental running test on a treadmill (Lode Valiant, Groningen, The Netherlands) to determine VO_2_max. The protocol and criteria for reaching VO_2_max were the same as used previously by our group [24]. Briefly, the exercise protocol consisted of a 4-min warm-up period (2 min at 8 km·h^−1^ with 0% inclination and a further 2 min at the same speed with a 5% inclination). The speed was then increased to 10 km·h^−1^ for 1 min with the gradient kept at 5%. For each minute thereafter, speed increased by 1 km·h^−1^ with the gradient at 5% up to a maximal speed of 16 km·h^−1^. From this stage, only the inclination increased by 2.5% every minute until exhaustion. Breath-by-breath ventilation and gas exchange were continuously analyzed (Ergostik, Geratherm Respiratory, Bad Kissingen, Germany) during the exercise with the data averaged to 30 s for analysis.

### 2.6. Experimental Repeated Sprint Protocol

The experimental protocol was performed on a standard indoor athletic surface. All participants performed a 10-min warm-up, including light jogging and progressive runs with acceleration, that was followed by 5 min of self-selected dynamic stretching. Participants then performed 3 practice sprint starts over a maximal distance of 10 m, followed by a 5-min rest period. The experimental protocol consisted of 15 × 30 m sprints interspersed by 20 s of active recovery (slow walk back to the start line). We chose a sprint length of 30 m, because 30-m repeated sprints are a valid test that significantly correlates with high-intensity parts in a soccer game [34]. Each participant ran repeated sprints in a separate running corridor without possible interaction with other participants to avoid affecting the sprint times. The time of each sprint was automatically measured using a digital photocell technology Tci timer (Brower Timing Systems, Draper, UT, USA). Photocells were set at the start line, at 15 m (intermediate line), and 30 m (finish line). The photocells were set at 0.7 m above the surface to avoid a false trigger caused by the limbs. After each sprint, participants provided an RPE score [35].

### 2.7. HRW and Placebo Preparation and Intake Schedule

HRW was obtained in 420 mL plastic aluminum packs with a gas-tight cap (Aquastamina-R, Nutristamina, Ostrava, Czech Republic). According to the manufacturer, HRW was produced from drinking water that underwent chlorine removal and H_2_ infusion under high pressure directly into the water as previously described by Kajiyama et al. [36]. Aquastamina-R does not contain any supplements except H_2_. The placebo was obtained from the HRW manufacturer and was produced in a similar way as Aquastamina-R and packed in the same packs. The only difference between Aquastamina-R and placebo was that the H_2_ infusion was shut down. Because H_2_ is colorless, odorless, and tasteless [15], it was not possible to distinguish HRW from placebo using the human senses. The type of water (HRW/placebo) was indicated on the pack using different batch numbers. The details of the batch numbers were kept confidential by the manufacturer until the experimental part and statistical analysis were completed.

A total volume of 1260 mL of HRW or placebo was administered in four doses. HRW dosing was inspired by a study [37] where the maximum concentration of H_2_ in exhaled air was recorded 30 min after HRW administration and returned to baseline in 60 min. Therefore, the time of the first 420 mL dose was set at 120 min, the second 420 mL dose at 60 min, and the last two 210 mL doses at 15 and 5 min before the repeated sprints (Figure 1). The division of HRW into 2 × 210 mL was due to concerns that drinking 420 mL of HRW 5 min before sprints could cause stomach discomfort. At the same time, we wanted to deliver at least some H_2_ as soon as possible before the repeated sprints. Once the 420 mL pack was opened, HRW/placebo was immediately administered to the participant. From the third pack, 210 mL was poured into a glass with a line indicating 210 mL and drunk immediately. The pack was closed with an air-tight cap. The remaining 210 mL was consumed 5 min before the sprints. Chemical characteristics of the HRW and placebo were determined using the pH/ORP/Temperature-meter (AD14, Adwa Instruments, Szeged, Hungary). Concentration of the dissolved H_2_ was determined using H_2_Blue reagent (H2 Sciences, Henderson, NV, USA) using the manufacturer’s guidelines. HRW/placebo characteristics were as follows: pH = 7.9/7.7, oxidation-reduction potential = −652/+170 mV, temperature = 20/20 °C, and dissolved H_2_ concentration = 0.9/0.0 ppm. A total absolute dose of 756 μmol of H_2_ was administered to each participant. The dose relative to body mass was 10.2 ± 1.1 μmol kg^−1^. This HRW hydration protocol included a 1-week washout period similarly to previous HRW studies [24,28,30].

### 2.8. Capillary Blood Lactate Sampling

Capillary (fingertip) blood samples were collected to assess lactate level. Alcohol wipes were used to clean participant fingers before sample collection. A lancet was used to puncture the skin and the first blood drop was wiped off. The second drop was analyzed using a blood analyzer Lactate Scout+ (EKF Diagnostics, Cardiff, United Kingdom).

### 2.9. Statistical Analysis

We expected the effect size in this study to be medium (*d* = 0.8) based on the results of Botek et al. [30]. An a priori power analysis with respect to paired two-tailed t-test and parameters set to α = 0.05 and β = 0.20 was performed using G*Power version 3.1.9.7 (Heinrich-Heine-Universität, Düsseldorf, Germany). The minimum sample size was 15 participants. We recruited 16 participants to have the same number in the two branches of the crossover study (*n*_1_ = *n*_2_ = 8).

The normality of data was verified using the Kolmogorov–Smirnov test. The HRW effect on blood lactate concentration was evaluated using a paired *t*-test. Sprint times were evaluated using a two-way analysis of variance (ANOVA) for repeated measures with two factors (water and time) and interaction (water × time). When water factor or interaction was statistically significant, pairwise comparisons were performed using Fisher’s LSD post hoc test. Differences in means were also expressed using 95% confidence intervals (CI). The significance level was set at α = 0.05. The Holm–Bonferroni method [38] was used to control the family-wise error rate. The statistical level for the set of 15 Fisher’s LSD post hoc tests was adaptive, and the current level in an iterative procedure was calculated on the number of rejected null hypotheses. For example, when two null hypotheses were rejected at the same time, both *p*-values had to be below 0.05/2 = 0.025.

Data are presented as arithmetic mean ± standard deviation (SD). Cohen’s standardized difference (*d*) was calculated based on the formula *d* = (*m*_1_ − *m*_2_)/SDdiff, where *m*_1_, *m*_2_ are means to compare and SDdiff is the standard deviation of the difference scores. The magnitude of Cohen’s *d* was interpreted according to the following thresholds: 0.00–0.19 trivial, 0.20–0.49 small, 0.50–0.79 medium, and ≥0.80 large. Statistical analyses were performed using MATLAB with Statistics Toolbox R2020a (MathWorks, Natick, MA, USA).

## 3. Results

Raw data are available in Table S1. Participants in this study did not report any side effects or complaints about HRW. The normal distribution was not rejected for all studied variables (all *p* ≥ 0.34). Urine specific gravity measured at the beginning of the experimental protocol was not statistically different between HRW and placebo (HRW: 1.011 ± 0.007, placebo: 1.013 ± 0.008, CI: −0.007 to 0.003, *p* = 0.43, *d* = −0.20, small effect).

**Figure 2:**
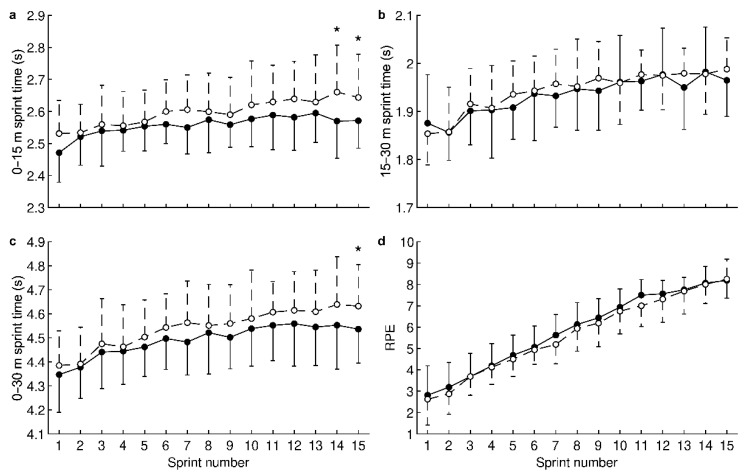
Effect of hydrogen-rich water on sprint time for the first half of the track (**a**); sprint time for the second half of the track (**b**); sprint time for the entire track (**c**); and rating of perceived exertion (**d**). ●—hydrogen-rich water; ○—placebo; ⋆—statistically significant (*p* < 0.025, Holm–Bonferroni method) difference between hydrogen-rich water and placebo. Values are presented as the mean and standard deviation.

For 0–15 m sprint times, ANOVA revealed a significant HRW effect (water factor: *p* < 0.001, time factor: *p* < 0.001, and interaction: *p* = 0.89). Pairwise comparisons showed that HRW significantly reduced times (Figure 2a) during the 14th sprint (HRW: 2.57 ± 0.12 s, placebo: 2.66 ± 0.15 s, CI: −0.15 to −0.03 s, *p* = 0.003, *d* = −0.74, medium effect, improvement of 3.4%) and the 15th sprint (HRW: 2.57 ± 0.09 s, placebo: 2.64 ± 0.13 s, CI: −0.13 to −0.01 s, *p* = 0.018, *d* = −0.59, medium effect, improvement of 2.7%). For 15–30 m sprint times (Figure 2b), no significant HRW effect was revealed (water factor: *p* = 0.066, time factor: *p* < 0.001, and interaction: *p* = 0.92).

For 0–30 m sprint times, a significant HRW effect was revealed (water factor: *p* < 0.001, time factor: *p* < 0.001, and interaction: *p* = 0.99). HRW significantly reduced times (Figure 2c) during the 15th sprint (HRW: 4.54 ± 0.14 s, placebo: 4.63 ± 0.17 s, CI: −0.18 to −0.01 s, *p* = 0.021, *d* = −0.58, medium effect, improvement of 1.9%). For RPE, a significant HRW effect was revealed (water factor: *p* = 0.014, time factor: *p* < 0.001, and interaction: *p* > 0.99). However, pairwise comparisons did not show a significant difference between HRW and placebo (all *p* ≥ 0.082, Figure 2d). On average, RPE with HRW was 0.2 higher compared to placebo, indicating no clinically significant effect.

Blood lactate concentration measured immediately after the repeated sprint test was not significantly different between HRW and placebo (HRW: 10.4 ± 3.2 mmol L^−1^, placebo: 10.4 ± 2.5 mmol L^−1^, CI: −1.2 to 1.1 mmol L^−1^, *p* = 0.93, *d* = −0.02, trivial effect).

## 4. Discussion

To the best of our knowledge, this is the first study to show an ergogenic effect of pre-exercise HRW consumption on repeated sprint performance. The primary findings of the present study were as follows: (a) a significantly faster 0–30 m sprint time (improved by 1.9%) for HRW compared to placebo during the last sprint; (b) for the 0–15 m distance, the 14th, and 15th sprints were significantly faster for HRW compared to placebo, representing improvements of 3.4% and 2.7%, respectively; (c) no significant differences between HRW and placebo were found for post-exercise blood lactate concentration or RPE.

The most interesting finding in this study was the significant dissociation in times (~2.5 s) for the last two 0–15 m repeated sprints between HRW and placebo. Research has suggested that during this type of repeated sprint protocol, there starts to be a reliance on aerobic metabolism that gradually counteracts the contribution of anaerobic glycolysis [4,9]. In relation to this, it is important to acknowledge that muscle CrP rephosphorylation, in contrast to ATP synthesis, is solely an oxygen-dependent process, which takes place in the mitochondria within the CrP shuttle [39], and post-exercise CrP replacement is linked, among other factors, with the current mitochondrial ATP pool [16]. In this regard, it was recently reported that H_2_ application induced an increase in mitochondrial membrane potential, oxygen consumption, and cellular ATP, indicating stimulated OXOPHOS and mitochondrial ATP production [26]. More recently, Gvozdjáková et al. [25] demonstrated an increase in mitochondrial respiration rate and ATP production in isolated mitochondria in response to H_2_ administration. However, whilst mitochondria are a major source of cellular energy, they are also significant producers of ROS/RNS that causes oxidative stress and peripheral fatigue [17,40]. In the context of repeated sprint exercise, it was recently suggested that excessive levels of ROS/RNS may cause a reduction in both mitochondrial efficiency and ATP production [16]. Research has shown that increased levels of oxidative stress can result in decreased muscle performance. Specifically, oxidative stress following 60 acute maximal eccentric actions of the elbow flexors led to a decline in their power output [41]. Since H_2_ has strong and selective antioxidant properties that protect against the negative effects of excessive ROS/RNS [20,22], it is possible that the H_2_ ingested as HRW in the present study may have played a role in the underlying mechanism that maintained mitochondrial efficiency and peak muscle power output in later stages of the repeated sprints.

Da Ponte et al. [33] also demonstrated an antifatigue effect of HRW intake during intermittent exercise. However, there are differences in methodology between the current study and that of Da Ponte et al. [33], whereby cyclists were involved, and the 30 min intermittent cycling consisted of 10 blocks. Each block lasted 3 min and consisted of 90 s at an intensity of 40% VO_2_max, 60 s at intensity of 60% VO_2_max, a 16 s maximal sprint and 14 s active recovery. The study [33] reported a 7.4% attenuation in the decline of relative peak power output particularly from the 6th to the 9th of 10 blocks with HRW supplementation. Based on previous and the current study findings, we feel that an acute pre-exercise HRW supplementation may be used as a novel antifatigue strategy that can prevent or reduce the decrement in running performance, especially during the later stages of repeated sprints. We propose that H_2_ most likely enhances mitochondrial respiratory efficiency, resulting in increased mitochondrial ATP production and CrP rephosphorylation as the key metabolic sources for muscle contraction within 2–3 s of maximal exercise.

However, our results also indicate no ergogenic benefit from H_2_ on running performance in the first thirteen 30 m sprints. In relation to this, it should be noted that the onset of sprint exercise was associated with initially low mitochondrial respiration and insufficient ATP production to meet the current ATP demands of working muscles [42], and this situation resulted in a significant activation of anaerobic ATP resynthesis within 2–3 s [4]. Dawson et al. [9] reported that only 70% of CrP was restored within 30 s of recovery after a single 6-s sprint. Based on this finding and only allowing 20 s of recovery between sprints, we feel that the antifatigue effect of H_2_ for the 15 m distance was more likely counteracted by the strong activation of anaerobic glycolysis during the second 15 m distance. This occurred because of the gradual withdrawal of the initial ATP-CrP muscle stores due to insufficient CrP recovery time between the subsequent sprints and a still marginal contribution of mitochondrial respiration to ATP resynthesis. Related to this explanation, the high post-exercise blood lactate concentration (10.7 mmol L^−1^) in this study indicated a significant anaerobic contribution, causing H^+^ accumulation and a subsequent decline in pH [43]. This decline in pH would inhibit CrP recovery [44] and result in a decline in repeated sprint performance [4,8]. In contrast to previous studies, intake of HRW did not impact RPE over the sprint protocol, with perception of effort increasing linearly and at the same rate in both conditions [24].

HRW significantly enhanced performance by 1.9% over the last 15 × 30 m sprints. At the end of the total sprint distance of 450 m, this meant that ingesting HRW allowed a 57 cm lead for a 30 m sprint. Placed in the context of a soccer game, this maintenance of repeated sprint ability would bring an advantage in the game. H_2_ itself is a safe and non-toxic substance, and oral ingestion HRW represents an easy, practical, and effective method of H_2_ administration [15,22]. Therefore, we conclude that HRW represents an interesting supplement for professional soccer players.

There are some limitations associated with this study, as follows. (a) The dosage of H_2_ was constant per participant and was not adjusted to body mass. (b) Sprint times were not measured using a system with several photocells, which would allow a more detailed assessment of the acceleration phase in repeated sprints. (c) Blood lactate concentrations were not analyzed in duplicate. (d) The oxidative stress response and oxygen consumption during the repeated sprints were not determined. This information may be helpful for a deeper understanding of how H_2_ may affect physiological responses during exercise.

## 5. Conclusions

The results suggest that intermittent, acute, pre-exercise HRW consumption with a total dose of 1260 mL caused an approximate 3.1% and 1.9% gain in 15 and 30 m sprint performance, especially during later stages of an experimental 15 × 30 m sprint protocol in professional soccer players.

## Data Availability

The data presented in this study are available in Supplementary Material.

## Supplementary Materials

The following supporting information can be downloaded at: https://www.mdpi.com/article/10.3390/nu14030508/s1, Table S1: Raw data.
